# A new magnetic melt spinning device for patterned nanofiber

**DOI:** 10.1038/s41598-021-88520-0

**Published:** 2021-04-26

**Authors:** Kai Zhang, Wu Zhao, Qingjie Liu, Miao Yu

**Affiliations:** 1 School of Mechanical Engineering, Sichuan University, Chengdu, 610065 China; 2 Sichuan Aerospace Vocational College, Chengdu, 610100 China; 3 Innovation Method and Creative Design Key Laboratory of Sichuan Province, Chengdu, 610065 China

**Keywords:** Engineering, Materials science

## Abstract

The size and morphology of nanofibers directly determine their application scope and performance, while regular patterned fibers further demonstrate their superior performance in the field of sensors and biomaterials. Melt electrospinning enables controlled deposition of fibers and is currently one of the most important means of preparing patterned fibers. However, due to the existence of high-voltage electric field, melt electrospinning has safety problems such as partial discharge and electric field breakdown, coupled with the charge rejection on the fiber surface, which seriously affects the positioning deposition of fibers and makes it difficult to obtain regular patterned fibers, greatly limiting the application areas and application effects of patterned fibers. Therefore, the improvement and innovation of the spinning process is particularly urgent. Based on material-field model and contradiction matrix of TRIZ theory, the problems of melt electrospinning device are systematically analyzed. The technical conflicts are solved by the inventive principles. A three-dimensional mobile magnetic melt spinning device model is constructed, a magnetic spinning test prototype is developed, and the prototype performance and influencing factors are studied by fiber morphology. The results show the following: (1) Replacing electrostatic fields with permanent magnetic fields can fundamentally avoid safety hazards such as electric field breakdown. (2) The magnetic field force on the molten polymer fluid can generate enough stretching force to overcome the surface tension and form fibers. (3) The fibers are deposited without a whipping instability phase similar to the electrospinning process, allowing easy preparation of regular patterned fibers. (4) The planar motion of the collector creates additional stretching effect on the fibers, which can further reduce the fiber diameter. (5) In magnetic spinning, no external high-voltage power supply is required, enabling the portability of the device. The results of this paper can provide a new method for preparing nanofibers with patterned morphology.

## Introduction

Nanofibers, in a broad sense, refer to ultrafine fibers with a diameter of less than 1000 nm. They have extremely high specific surface area and porosity, light weight and flexibility, etc., and can be modulated in fiber structure on micro and nano scales, occupying an irreplaceable position in the fields of composite materials, catalysis and sensing^1,2^. In recent years, a variety of methods for preparing nanofibers have emerged, such as phase separation, self-assembly, mechanical stretching and template synthesis. Due to the technical complexity and high manufacturing cost, as well as low production efficiency and fiber quality, the above fabricating processes limit their wide application in the preparation of nanofibers^3–5^.

Electrospinning or electrospun, first proposed by Fornhals A. is a simple technique in structure and owns high production efficiency^6^. After continuous improvement and development by many researchers, electrospinning has become the most commonly used method for the preparing nanofibers^7–11^. Electrospinning techniques mainly include far-field electrospinning (FFES)^12,13^, near-field electrospinning (NFES)^7,14^, and melt electrospinning (MES)^15,16^ etc. However, these methods have their own advantages and disadvantages. According to the different requirements and application scenarios of nanofiber fabrication, they have been widely studied and applied in a certain period of time, and have promoted the development of nanofiber preparation technology to a certain extent, and achieved good practical effects.

With the development of bionics and tissue engineering, new and higher requirements have been put forward for the fineness and regular arrangement of nanofibers^17,18^. The FFES technique has been widely used in preparing nanofiber efficiently, nevertheless the occurrence of whipping instability (bending instability) stage in spinning process and the residual charge repulsive effect between the deposited fibers on the collector and the subsequently depositing fibers. These problems make the preparation of regular patterned fibers extremely difficult. In addition, the ultra-high DC voltage (10–50 kV) applied during the spinning process increases the safety hazards such as electric field breakdown, partial discharge, high energy consumption and solvent pollution of the environment are also urgent problems to be solved. To obtain regular patterned fibers, researchers developed NFES technique, which successfully avoids the whipping instability stage by shortening the spinning distance to millimeter level and achieves controlled deposition of fibers more easily, but electric field breakdown, solvent contamination and electrical energy consumption remain unsolved. In addition, polymer droplets are prone to dropping to destroy the fiber morphology on collector due to the short spinning distance in NFES. The MES technique, being explored to solve the above problems, prepares fibers by heating the polymer to a molten viscous flow state, avoiding the use of organic solvents, and is an environmentally friendly spinning process. The higher viscosity and surface tension of the polymer melt further suppresses the whipping instability and allows easier access for regularly patterned fibers. However, MES still uses a high-voltage electric field as power source, and problems such as electric field breakdown and charge rejection remain. In addition, the MES distance is short and the electric field force is not sufficient to stretch the fibers, and the prepared regular fibers are difficult to reach the nanometer scale. Therefore, it is of great practical importance to study safe, effective, convenient and green methods for the preparation of regular patterned fibers. In recent years, researchers have improved and optimized the preparation process of patterned fibers by applying auxiliary electric and magnetic fields, such as Yang et al.^19^ introduced a magnetic field in an electrospinning device to assist in the regular arrangement of nanofibers. Tokarev et al.^20^ proposed a spinning method using magnetic field force to stretch ferromagnetic fluid into fibers, but failed to prepare regular patterned fibers. Currently, this technology is still in the technology exploration stage and has not been made into a spinning device.

Aiming at the problems of pattern fiber preparation, poor safety, solvent pollution and high energy consumption existing in the existing spinning process, this paper developed a magnetic melt-spinning (MMS) device based on MES technology and combining the advantages of the above methods. During MMS process, permanent magnetic field is selected as the spinning energy source instead of high-voltage electric field. And the heating and propulsion device of melt spinning is improved for heating, mixing, storing and supplying polymeric magnetic fluid. A three-dimensional mobile collecting platform is constructed to assist in stretching and precisely depositing fibers through controlling the moving trail and speed, and in this way, nanoscale patterned fibers are prepared. Compared with the electrospinning process, the MMS process requires no high-voltage electric field, no whipping instability stage, no charge rejection problem, and no organic solvent contamination. In addition, the MMS technology has good controllable deposition, high safety, and no pollution, and regular patterned fibers can be prepared conveniently.

## Melt electrospinning

**Figure 1:**
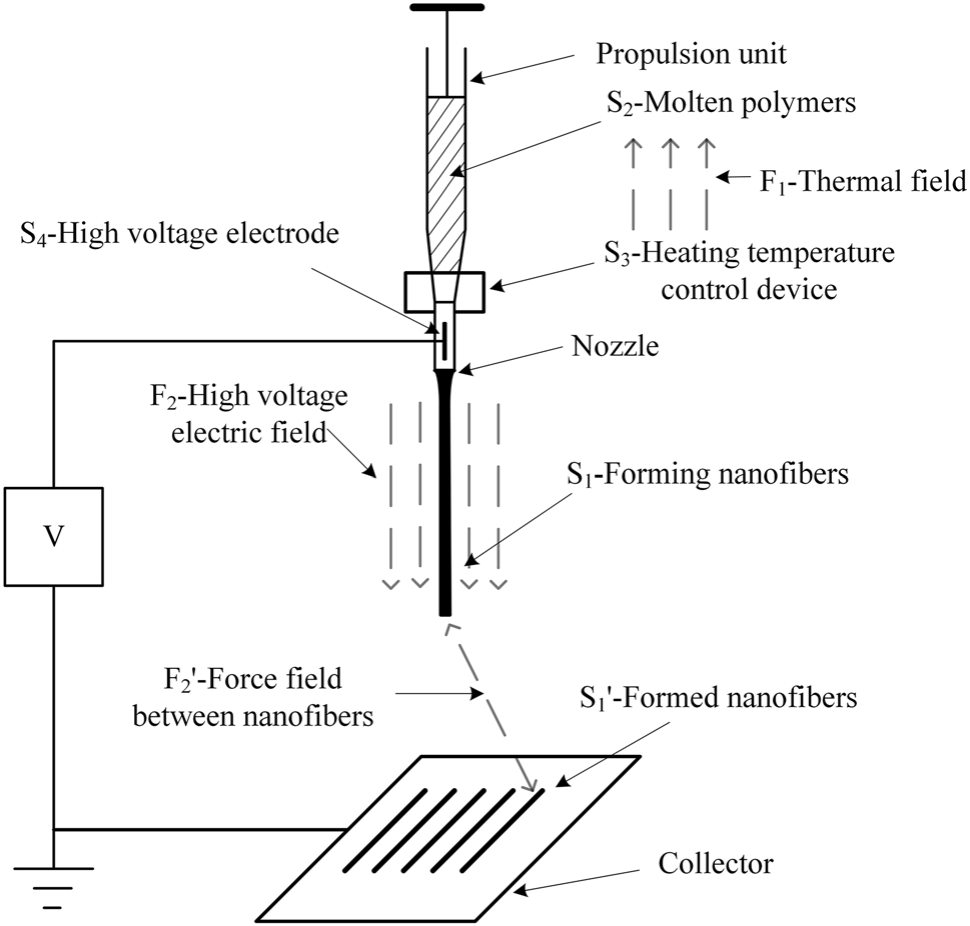
Schematic diagram of MES device.

MES technology is based on NFES technology and generally uses resistive heating to heat the polymer above its melting point to obtain a polymer melt. The molten polymer fluid is subjected to electric field forces, gravity and surface tension in an electrostatic field, and when the electric field forces and gravity overcome the surface tension, the molten fluid is stretched into fibrous filaments that are deposited toward the collector^21^. The MES process unit is shown in Fig. 1 and consists of a polymer propulsion unit, a heating temperature controlling device, a high voltage electric field, and a collector.

MES has the following advantages: (1) It is an environmental friendly fiber preparation process without using organic solvents; (2) The microfiber/nanofiber device with smooth and continuous surface can be prepared; (3) In the spinning process, the viscosity of the molten polymer fluid is higher (compared to polymer solution), which reduces the whipping instability and is beneficial to obtain regular pattern fibers. However, there are still some shortcomings: (1) MES still uses high voltage electric field as energy source, which does not fundamentally eliminate problems such as whipping instability, charge repulsion and electrical safety; (2) The diameter of fiber prepared by MES is coarse.

## Device design of MMS

### Problem analysis of MES

**Figure 2:**
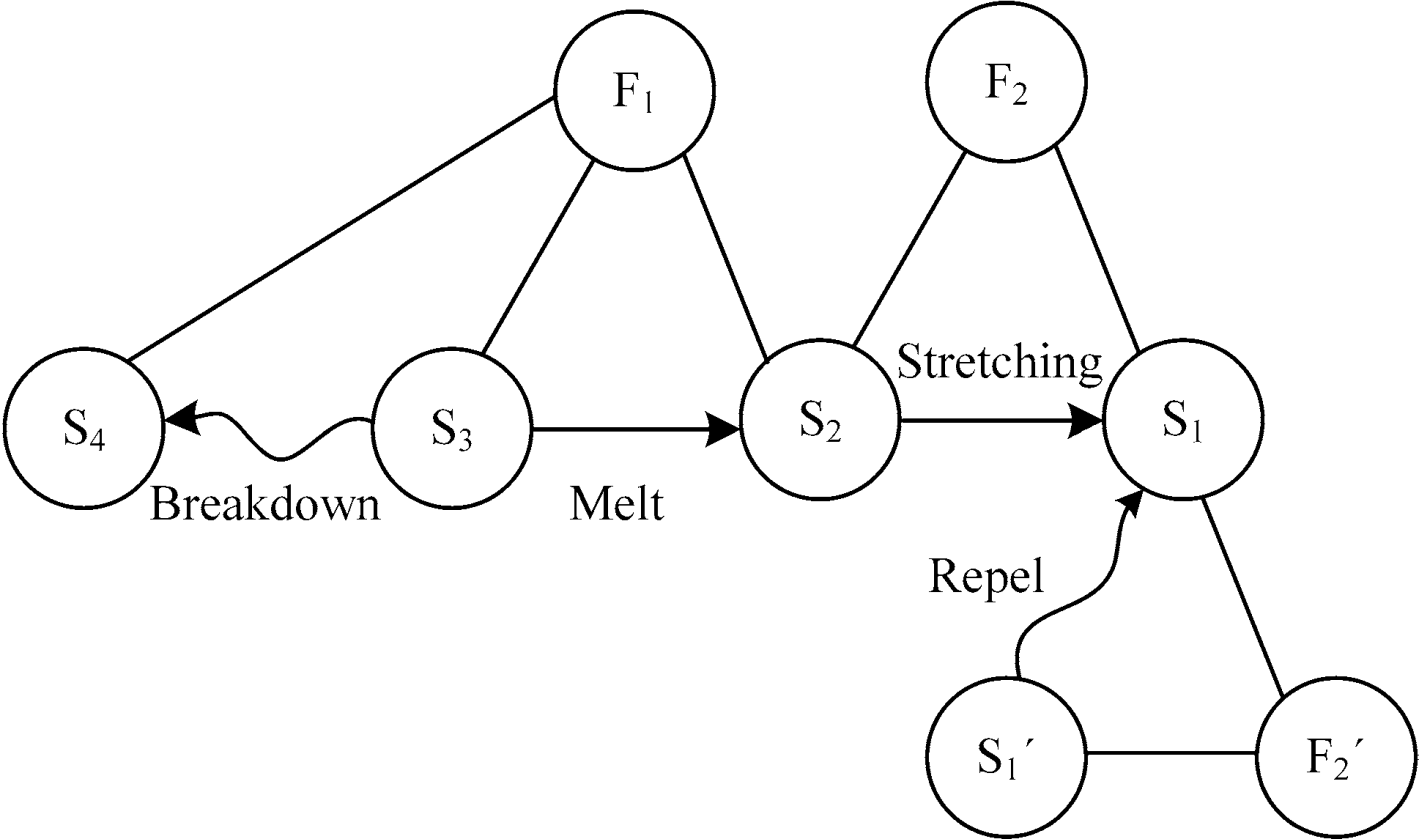
Material-field model of MES.

TRIZ theory is an effective method for resolving conflicts and eliminating contradictions. Among them, the material-field model analysis method is a method used to establish a functional model linked to the problem of an existing system or a new technological system, and in the process of problem solving, the corresponding general and standard solutions can be found according to the problem described by the material-field model^22–24^. To address the problems of MES, a melt spinning design solution strategy based on the material-field model and conflict resolution theory is proposed to eliminate the whip instability in electrospinning to obtain regular patterned fibers with fine particle size. The MES system shown in Fig. 1 can be decomposed and represented by multiple material-field triangles modeled, and the obtained material-field model is shown in Fig. 2.

The process of solving this problem by the material-field model analysis is as follows.

(1) Specify the substances: S_1_-forming nanofibers, S_1_′-formed nanofibers, S_2_-molten polymer, S_3_-heating temperature control device, S_4_-high voltage electrode.

(2) Determine the fields: F_1_-thermal field, F_2_-high voltage electric field, F_2_′-force field between nanofibers.

The analysis of the material-field model shows that: (1) the heating temperature control device S_3_ keeps the molten polymer S_2_ always in the molten state under the action of the thermal field F_1_ (effective effect); (2) the high-voltage electrode is connected to the melt nozzle or needle part, and the electric heating or other direct heating methods are used, which will easily lead to the breakdown of the high-voltage electrode S_4_ (harmful effect). The melt spinning distance (the distance between the collector and the nozzle) is continuously adjustable, when the spinning distance is far: (3) the jet stream of molten polymer S_2_ is continuously stretched under the action of high-voltage electric field F_2_ until it is pulled into nanofiber S_1_ (effective effect); (4) the nanofiber S_1_ formed in the high-voltage electrostatic field is homogeneously charged, and the nanofibers repel each other under the action of inter-nanofiber force field F_2_′, and the final formed nanofiber S_1_′ is difficult to have a good morphology (harmful effect). In addition, the fibers can cure prior to deposition, which affects the positional deposition performance, and requires higher spinning voltages, increasing the risk of partial discharge and breakdown. When the spinning distance is shortened, the fibers have not reached the whip instability stage has been stacked on the collector, so that the fibers have not had time to pull the fine stacked shape, which eliminates the harmful effects of F_2_′, can get a regular arrangement of fibers, but at the same time lead to F_2_ does not play a corresponding stretching effect, the obtained fiber particle size is coarse. That is, MES exists between the contradiction of fiber size and alignment rules, it is difficult to get both fine particle size and regular arrangement of the fiber pattern.

This system is a complete material-field model of harmful effects. The main problems are the poor safety of the spinning system and the irregular shape of the collected nanofibers or the coarseness of the collected fibers that do not reach the nanoscale.

### Problem solving of MES

The analysis of the forming process and the material field model of MES revealed that: (1) Melt electrospinning require applying high voltage between nozzle and collector, and the melt polymer undergoes electrical filed stretching to form fiber to deposit on the collector as charges were inject into polymer jet. Under the circumstance, local corona discharge or breakdown often occurred when the applied voltage reached the limit^11^. (2) The newly formed nanofibers are homogeneously charged, and the collected nanofibers are irregular in shape due to the repulsion of the charges; the high voltage can make the nanofibers being formed have a greater force in the axial direction, which makes the repulsion effect between the nanofibers less obvious, so that the nanofibers with better shape can be collected, but the power consumed is too large and the safety risk is greater.

For the above-mentioned harmful material-field models, the effects of harmful effects can be eliminated by introducing new physical fields or by inventive principles^22,24^. Using the TRIZ theory of technical conflict resolution to analyze the above problems^25^: (1) The temperature of the melt must be guaranteed, and "temperature" is the parameter to be improved, which corresponds to the general technical parameter "22-temperature"; the safety is affected by the breakdown of the circuit or high-voltage electrode, and "safety" is the parameter to be deteriorated, which corresponds to the general technical parameter "37-safety". "Safety" is the parameter to be deteriorated, corresponding to the general technical parameter "37-safety". A query of the TRIZ theory conflict matrix yielded 5 recommended inventive principles: 24 (with the help of intermediaries), 1 (segmentation), 3 (local mass), 35 (change of physical or chemical parameters), 28 (substitution of mechanical systems). (2) Well shaped nanofibers need to be obtained, "well shaped nanofibers" is the characteristic to be improved, corresponding to the general technical parameter "9-shape"; loading higher voltage causes excessive energy consumption, "increased energy consumption" is the characteristic to be deteriorated, corresponding to the general technical parameter "16-energy consumed by moving objects". A query of the TRIZ theory conflict matrix yielded 8 recommended inventive principles: 3 (local mass), 14 (surfacing), 28 (substitution of mechanical systems), 2 (extraction), 24 (with the help of intermediaries), 35 (change of physical or chemical parameters), 6 (diversification), 34 (discard or regeneration). The inventive principle 28 (substitution of mechanical systems) was selected after comparative analysis. Using a strong magnet instead of high-voltage electricity and adding ferromagnetic particles to the polymer melt to make it a magnetic fluid, the magnetic field generated by the strong magnet is used to spin the magnetic fluid, which retains the advantages of MES and solves two pairs of technical conflicts: (i) the high-voltage circuit is eliminated, the temperature of the melt will not be affected, and the safety of the melt temperature and preparation process can be ensured at the same time; (ii) the high-voltage electric field is eliminated and the magnetic field is used, which ensures the morphology of the patterned nanofibers obtained and does not increase the energy consumption.

**Figure 3:**
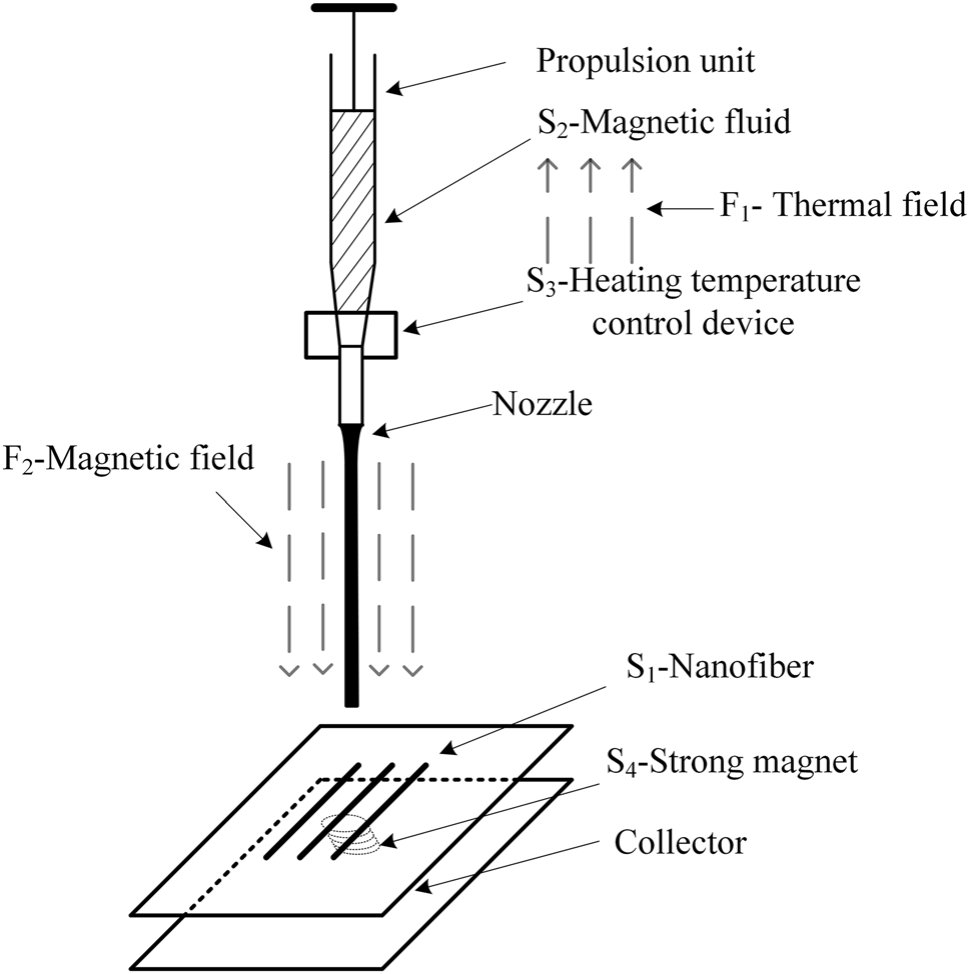
Schematic diagram of MMS device.

**Figure 4:**
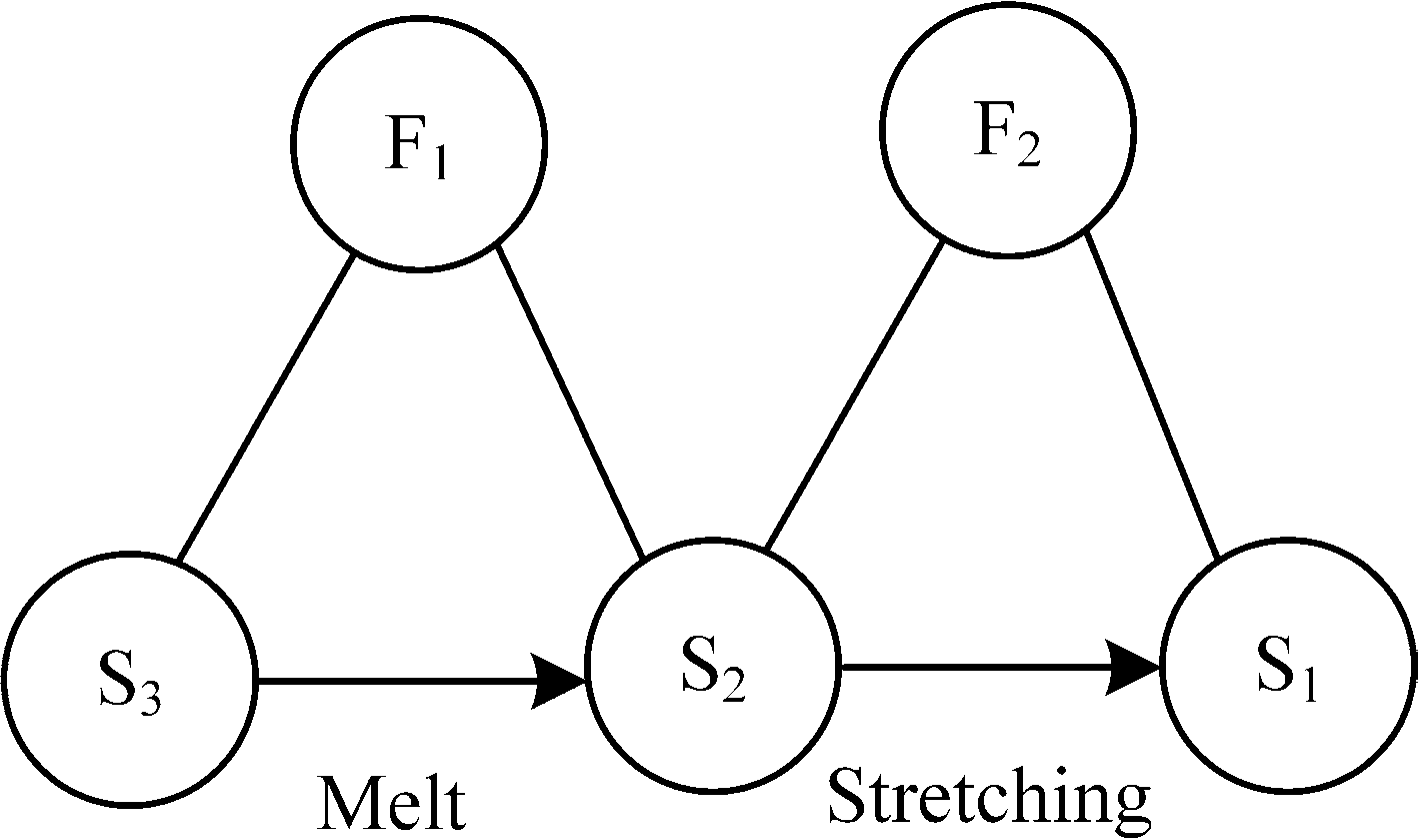
Material-field model of MMS.

A schematic diagram of the modified MMS device using the conflict matrix is shown in Fig. 3, and a model of the MMS material-field is shown in Fig. 4.

The MMS process is analyzed using the material-field model as follows.

(1) Specify the substances: S_1_-nanofiber, S_2_-magnetic fluid, S_3_-heating temperature control device, S_4_-strong magnet.

(2) Determine the fields: F_1_-thermal field, F_2_-magnetic field.

The analysis of the material-field model shows that: (1) the magnetic fluid S_2_ is always in the molten state under the action of the thermal field F_1_ through the heating temperature control device S_3_ (effective effect); (2) the jet stream of the magnetic fluid S_2_ is continuously pulled thin under the action of the magnetic field F_2_ until it is formed into nanofiber S_1_ (effective effect).

This system is an effective and complete material-field model, using the inventive principle, a strong magnet is substituted for high voltage electricity, and the MES device is improved, thus solving the problems of poor safety and irregular shape of the collected patterned fibers in the MES device.

### Device improvement of MMS

**Figure 5:**
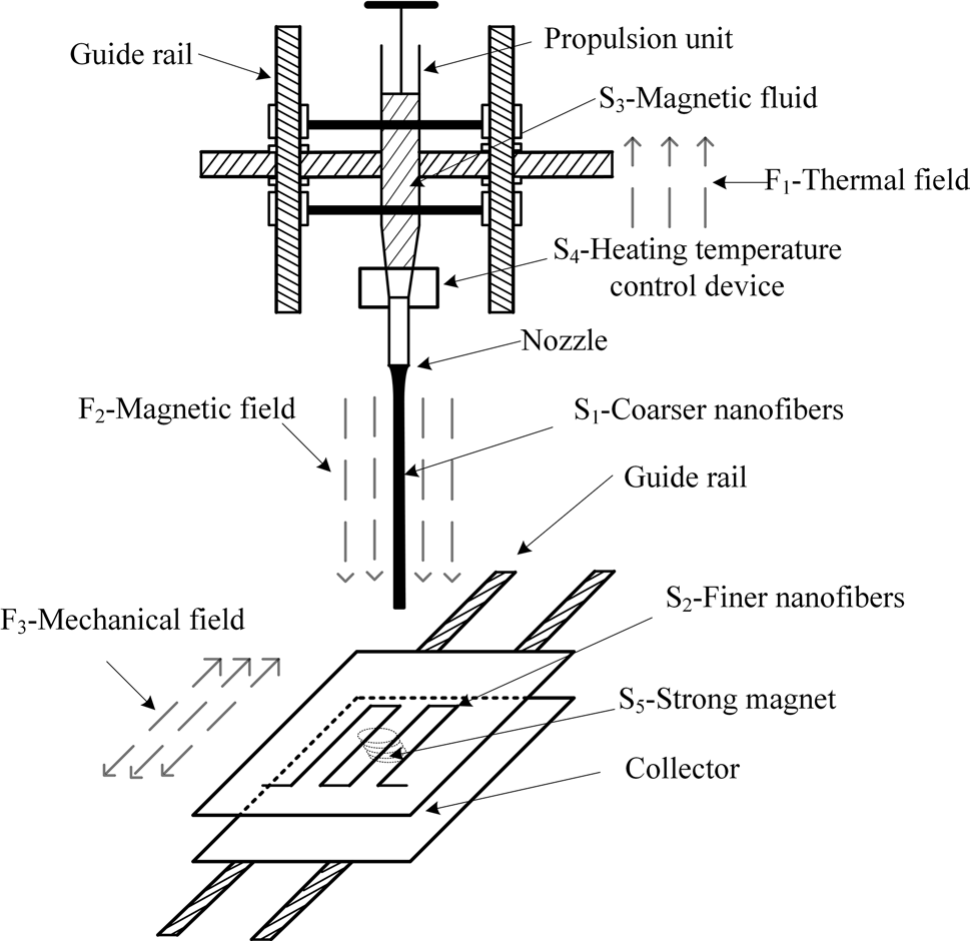
Schematic diagram of three-dimensional mobile MMS device.

In the problem solving session, the problems of poor safety and irregularity of the collected patterned fiber morphology in the MES device were solved, and the melt magnetic spinning device was obtained. However, the working platform of this device and the position of the magnetic fluid injection device are fixed, resulting in the prepared nanofiber morphology is almost constant, limiting the versatility and operational flexibility of the prepared nanospinning, and the prepared nanofibers are coarse and difficult to reach the nanometer scale. Some researchers have solved the problems of coarse nanofibers and low spinning efficiency by changing the fixed working platform into a rotating working platform^26^, but there is still a lack of research on how to form patterned fibers, which cannot meet the demand of neatly arranged nanofibers. Therefore, the MMS device shown in Fig. 3 is optimized to solve this problem by the three-dimensional movement between the magnetic fluid injection device and the working platform. Figure 5 shows a schematic diagram of the three-dimensional mobile MMS device after the optimization of the MMS device.

**Figure 6:**
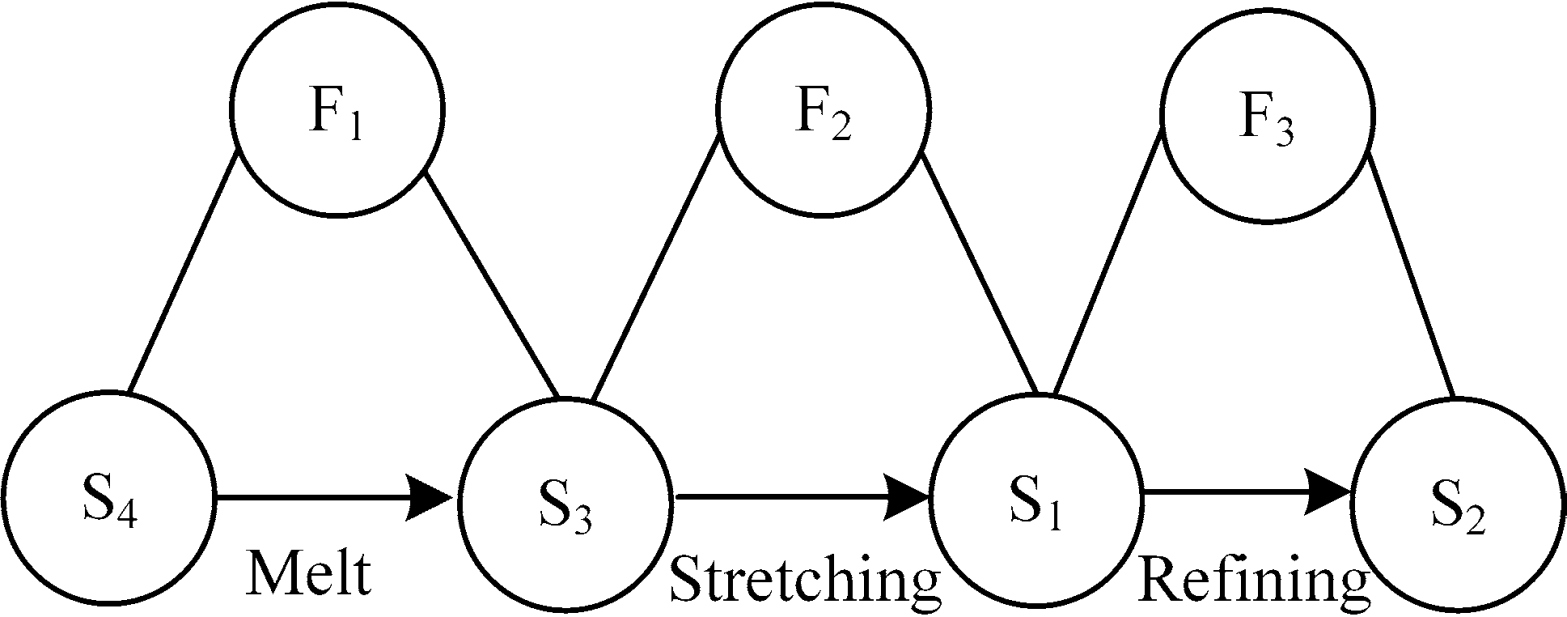
Material-field model of three-dimensional moving MMS.

The three-dimensional mobile MMS device spinning process is analyzed using the material-field model (shown in Fig. 6) as follows.

(1) Specify the substances: S_1_-coarser nanofibers, S_2_-finer nanofibers, S_3_-magnetic fluid, S_4_-heating temperature control device, S_5_-strong magnet.

(2) Determine the fields: F_1_-thermal field, F_2_-magnetic field, F_3_-mechanical field.

The analysis of the material-field model shows that: (1) the magnetic fluid S_2_ is always in the molten state under the action of the thermal field F_1_ through the heating temperature control device S_4_ (effective effect); (2) the jet stream of the magnetic fluid S_3_ is continuously pulled thin under the action of the magnetic field F_2_ until it is formed into the nanofiber S_1_ (effective effect); (3) the coarser nanofiber continues to be pulled thin under the action of the mechanical field F_3_ until it becomes the nanofiber S_2_ in a more ideal state (effective effect).

This system is an effective and complete material-field model. In this system, the original fixed magnetic spinning device is changed into a three-dimensional magnetic spinning device that can be moved up and down, left and right, and back and forth, by optimizing the melt magnetic spinning device for an effective complete object field model. This device can obtain finer nanofibers and can generate regular patterned fibers with uniform nanofiber particle size by constantly changing the relative positions of the magnetic fluid preparation device and the mobile table.

### Device details optimization

**Figure 7:**
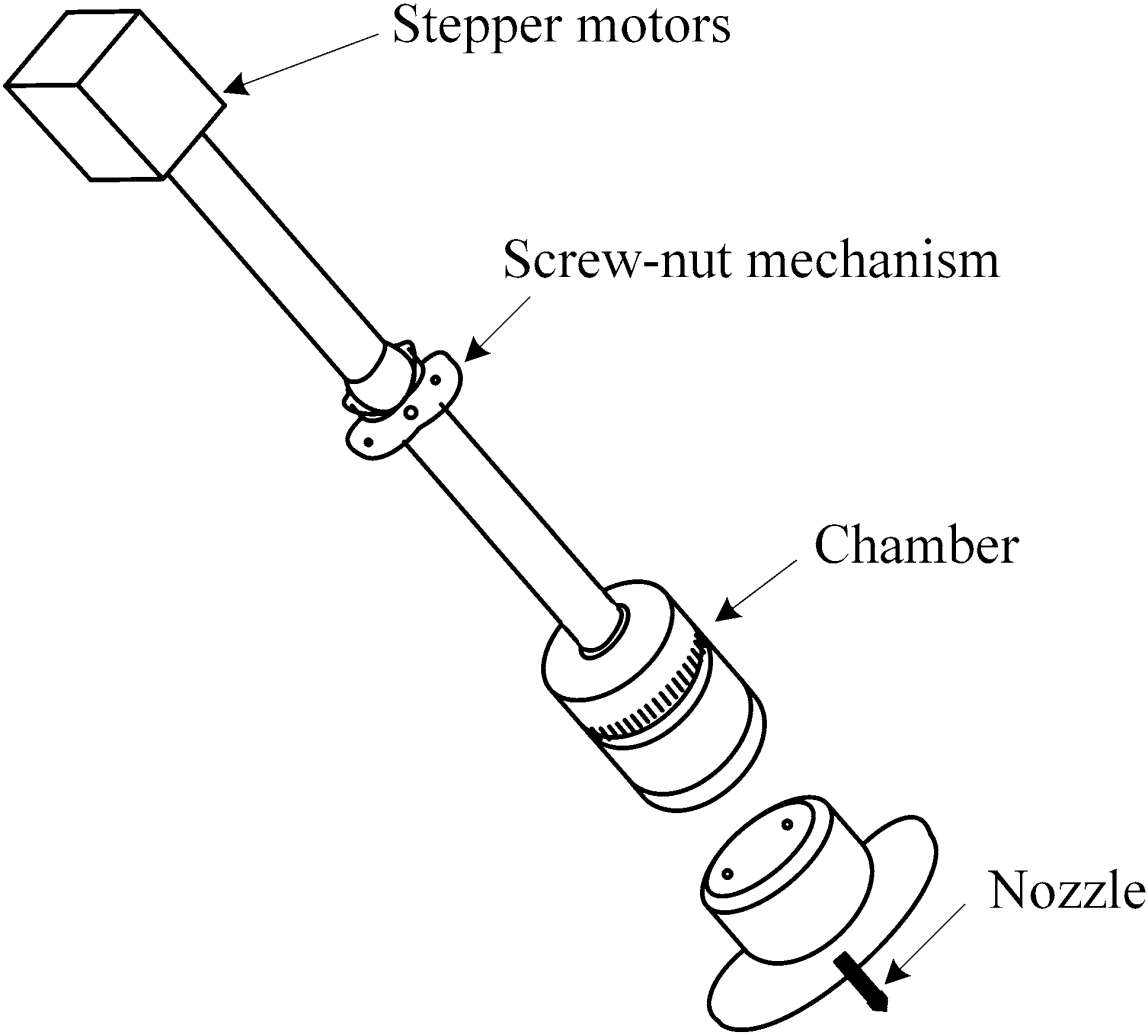
Magnetic fluid injection device.

In-depth analysis of the three-dimensional mobile MMS device shown in Fig. 5 found that the device also has some details of the problem. For example, when the magnetic fluid is extruded out of the capillary syringe little by little through the piston, it is difficult to control the extrusion force and the speed of the magnetic fluid outflow due to the manual extrusion operation, which makes the extruded magnetic fluid not uniform, thus leading to the inconsistent diameter of the prepared nanofibers. To address this problem, a stepper motor magnetic fluid extrusion device is designed in this paper, as shown in Fig. 7.

The stepper motor is connected to the screw nut mechanism, and the screw nut mechanism is connected to the pushing member so as to control the piston movement, and the piston movement is used to regulate the pressure acting on the magnetic fluid inside the chamber, thus making the magnetic fluid extruded more stably and uniformly, so as to prepare the fiber with uniform particle size, which solves the problems of the traditional feeding device that the feeding is not uniform and stable enough and the spinning effect is poor.

### Three-dimensional mobile MMS principle

**Figure 8:**
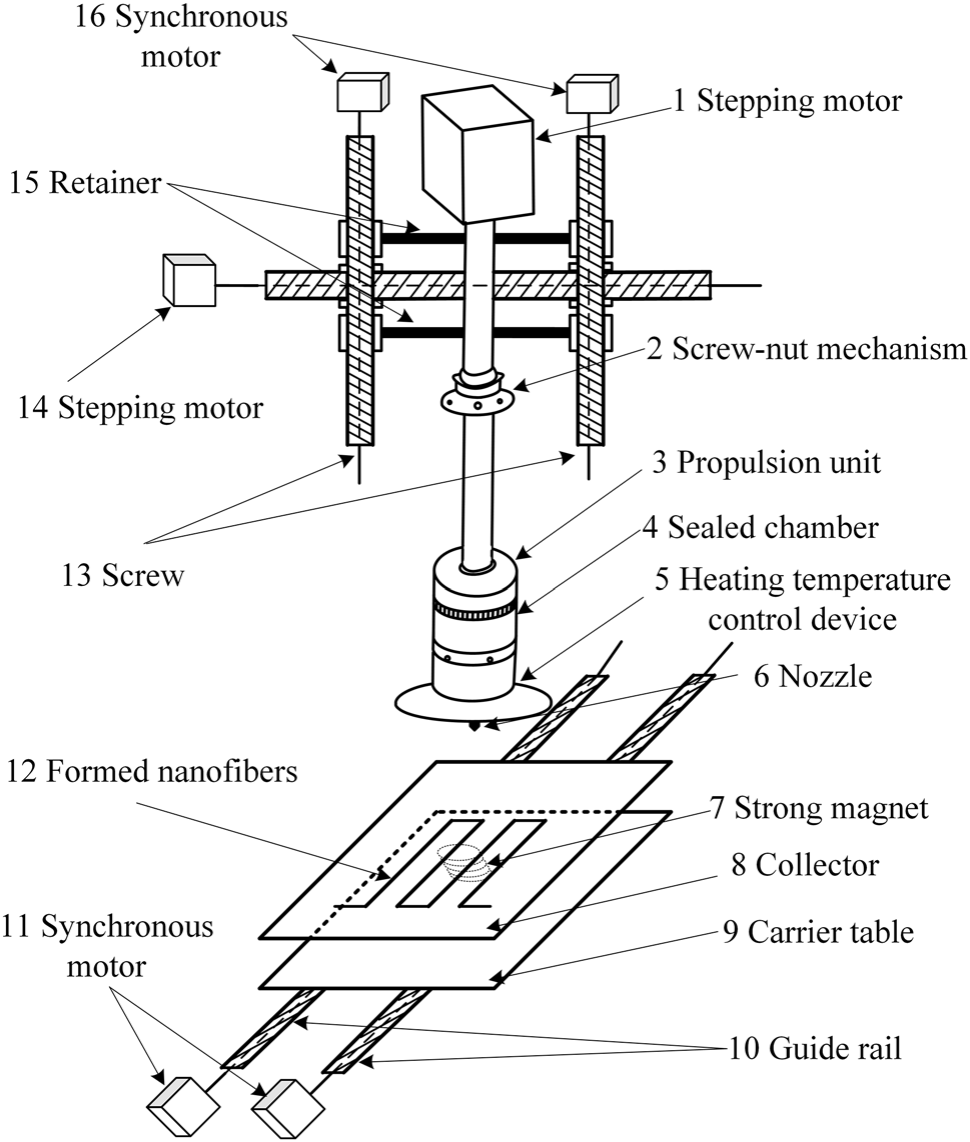
Three-dimensional mobile MMS device.

After further optimization of the magnetic fluid spinning device, a three-dimensional mobile MMS device was finally developed, the structure of which is shown in Fig. 8. The MMS device consists of four main parts: the magnetic fluid preparation device for producing magnetic fluid, the three-dimensional console for fixing the magnetic fluid preparation device, the collector located below the magnetic fluid preparation device, and the strong magnet located below the collector.

The spinning material is put into the sealed chamber 4, and then the magnetic fluid preparation device is fixed on the support of the three-dimensional console, and the relative position of the nozzle 6 of the magnetic fluid preparation device and the strong magnet 7 of the three-dimensional console is adjusted to the set initial position. By controlling the stepping motor 1, the propulsion device 3 is moved downward to the sealing chamber 4 under the action of the filament nut mechanism 2, and the pre-prepared magnetic fluid in the molten state is added inside the sealing chamber, which is always in the molten state under the control of the heating temperature control device 5. Under the further action of the propulsion device, the magnetic fluid inside the chamber is extruded from the nozzle under pressure to form a liquid droplet. The synchronous motor 11 is connected to the screw, which is connected to the guide rail to control the front and back relative position of the working platform and the nozzle, the stepping motor 14 controls the left and right relative position of the working platform and the nozzle, and the synchronous motor 16 controls the up and down relative position of the working platform and the nozzle. The relative position of the nozzle of the magnetic fluid preparation device and the strong magnet of the three-dimensional console can be freely controlled, and the droplet-shaped magnetic fluid formed according to the designed trajectory forms a liquid bridge under the action of the magnetic field of the strong magnet, and the liquid bridge is cured to form nanofiber filaments.

## Experiment

### Experimental materials and equipment

**Figure 9:**
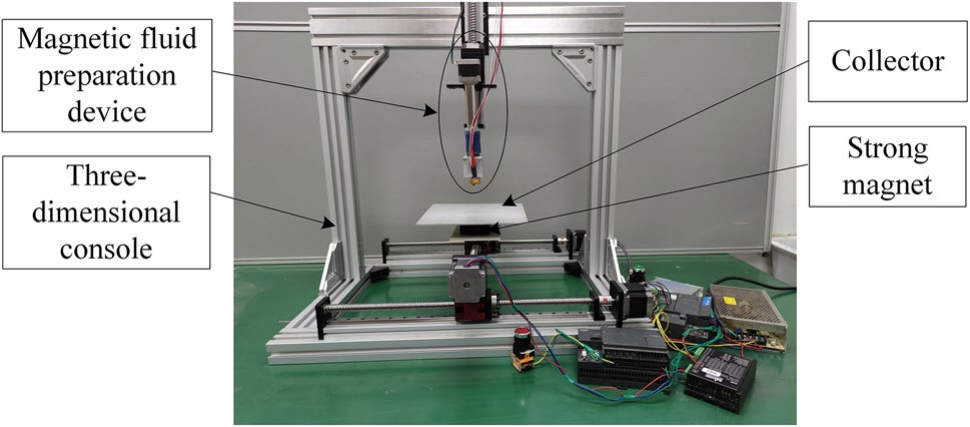
MMS test prototype.

Polystyrene (PS, MW = 300 kDa) was chosen as the raw material for polymer spinning, the average particle size of Fe_3_O_4_ magnetic nanoparticles was 50 nm, and the doping ratio of Fe_3_O_4_ in the polymer was 5%. The experimental equipment was a homemade three-dimensional mobile MMS test prototype shown in Fig. 9. The experimental instruments include a optical microscope (Zeiss Axio Lab, Germany), a scanning electron microscope (FEI Quanta 200, The Netherlands), an atomic force microscope (Bruke Multimode 8, Germany), and an X-ray diffractometer (X'Pert PRO, The Netherlands).

### Experimental method

PS and Fe_3_O_4_ nanoparticles were loaded into the magnetic fluid melt chamber separately, and the PS-Fe_3_O_4_ mixture in the molten state was obtained by heating to 210 °C. The propulsion device extruded the fluid from the nozzle at a speed of 0.4 ml/h to form droplets. The spinning distance is set to 5 mm, which is the distance between the nozzle and the collector. The collector is controlled by the moving platform and moves according to the set trajectory line in the range of 0–10 cm, with a moving speed range of 0–100 mm/s and a positioning accuracy of 0.01 mm.

### Analysis of experimental results

**Figure 10:**
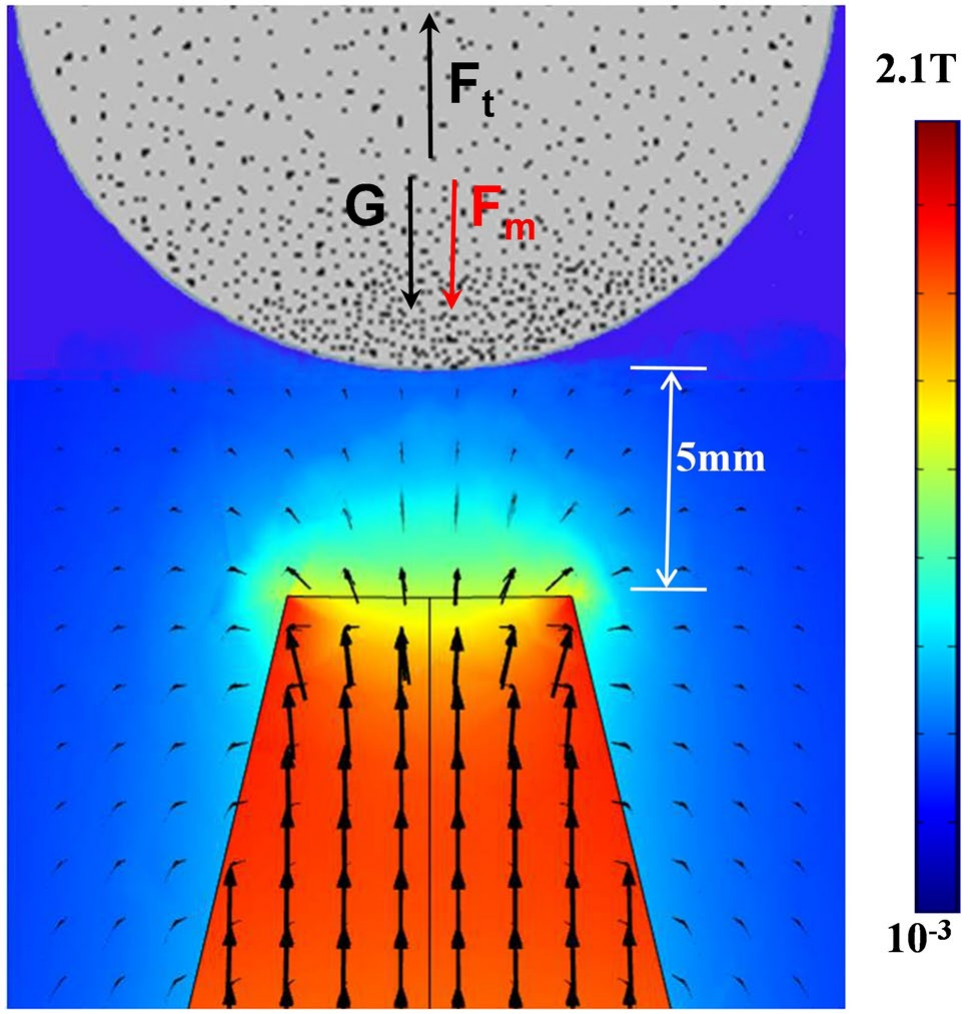
Magnetic spinning simulation diagram.

**Figure 11:**
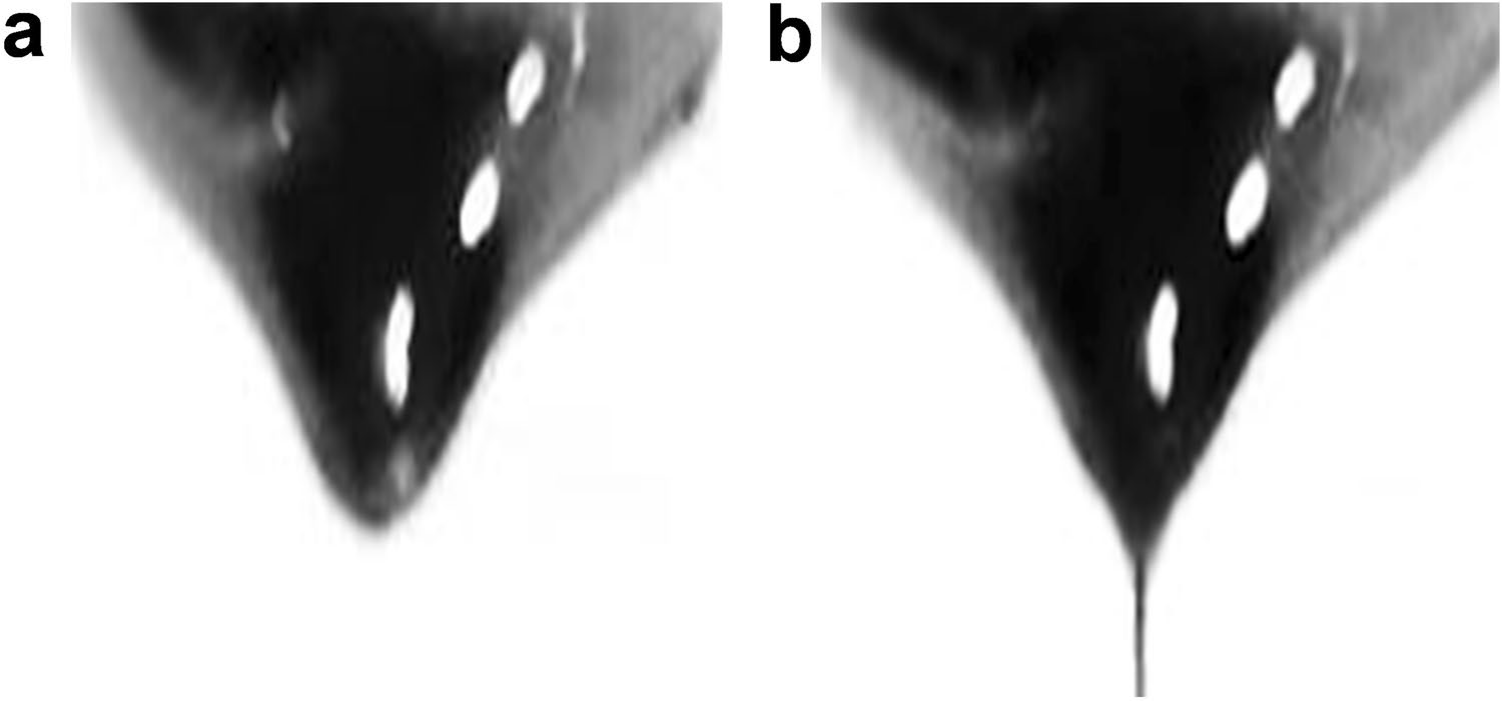
Magnetic droplets stretched by magnetic force to form fibers.

As shown in Fig. 10, the permanent magnet in the experiment generates a permanent magnetic field to the surrounding environment. As shown in Comsol simulation, the magnet generates a magnetic field of about 0.8–1.6 T (Tesla) in a region of about 5 mm outside the tip. Without magnetic field, the polymer droplet is mainly subjected to gravity (G) and surface tension (F_t_), when G ≤ F_t_, the droplet maintains its own shape and does not drip. When the magnetic field force is applied, the droplet is subjected to G, F_t_ and the magnetic field force (F_m_) at the same time. When G + F_m_ ≥ F_t_, i.e., gravity and magnetic field force overcome the droplet surface tension, the droplet is stretched to form a taylorcone (Fig. 11a) and then gradually forms a fibril (Fig. 11b), which is deposited onto the collector. At the same time, the collector makes a planar motion under the control of the set program, and the motion generates additional mechanical stretching force on the fibers, which further shrinking the fiber.

**Figure 12:**
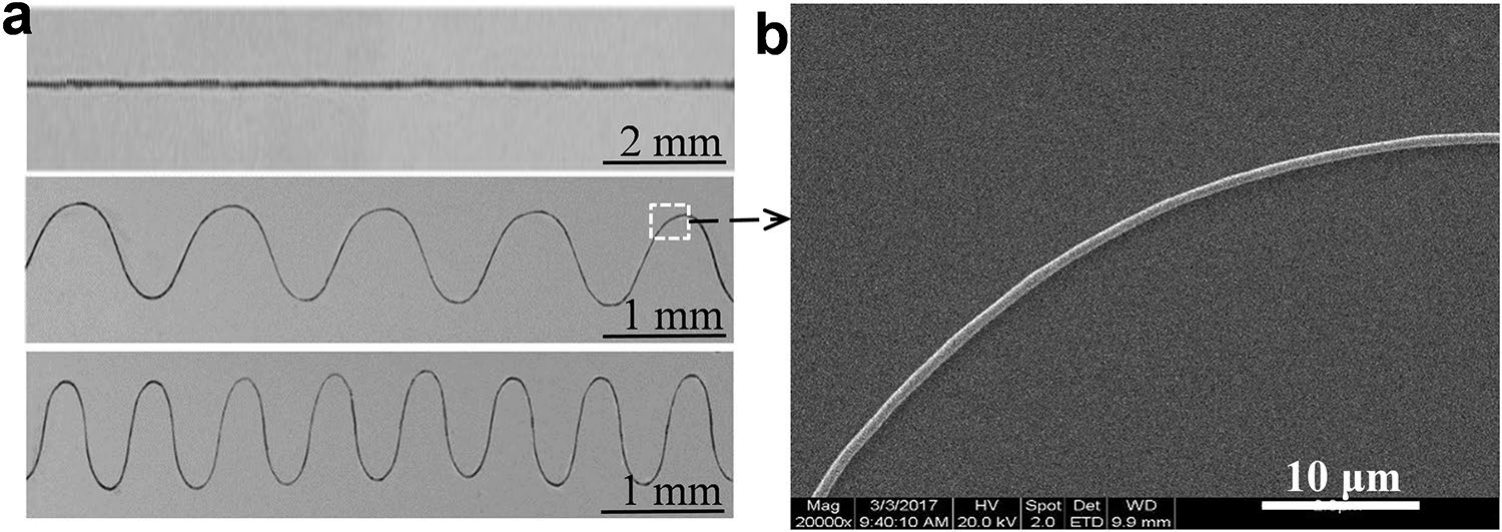
2D patterned fiber prepared by magnetic spinning and SEM image.

**Figure 13:**
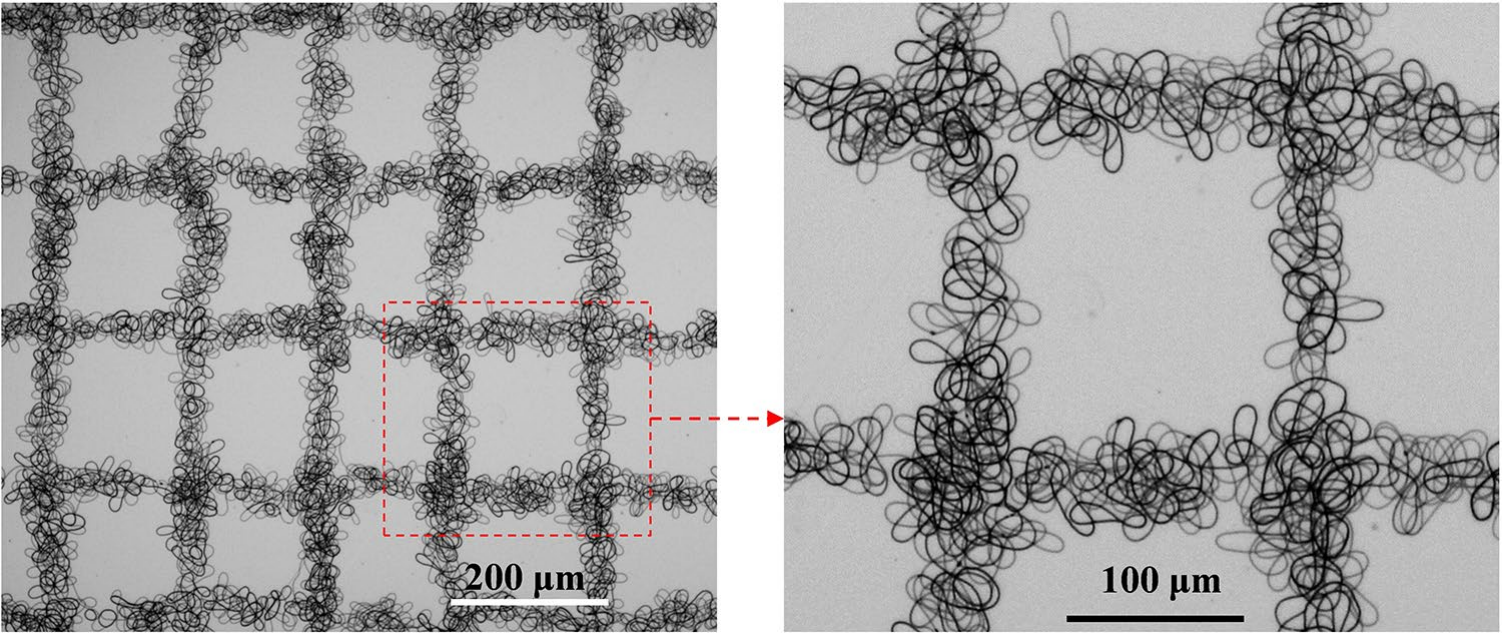
Patterned architecture with orthogonally laid fibrous strips.

Regular fiber from MMS were first characterized by scanning electron microscopy (SEM), as shown in Fig. 12a, when the collector moves in-plane with a linear trajectory at a speed of 10 mm/s, linear fibers with a particle size of approximately 10 μm are obtained. When the collector moves in a plane with a sinusoidal trajectory (speed of 20 mm/s), corresponding 2D sinusoidal pattern fibers are obtained. In addition, the morphology of the patterned fibers, including the frequency and amplitude of the waveform, can be changed by adjusting the frequency of the sinusoidal trajectory motion of the collector. As shown in Fig. 12b, the average particle size of the prepared sinusoidal fibers is about 900 nm. Besides, a patterned architecture has been fabricated through orthogonally laid fibrous strips with strip width of 50 μm and interval of 200 μm, as shown in Fig. 13. From the experimental results, it can be seen that patterned fibers with good morphology and uniform particle size can be prepared by this magnetic spinning process.

**Figure 14:**
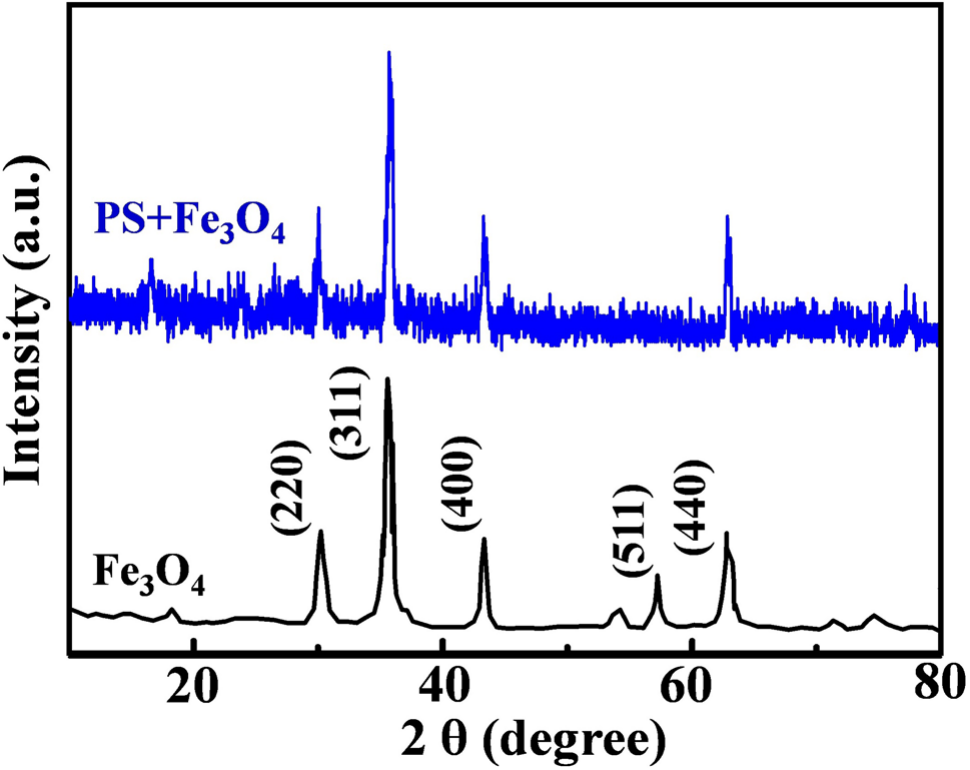
XRD patterns of PS and PS-Fe_3_O_4_ composites.

Figure 14 shows the XRD patterns of Fe_3_O_4_ and PS-Fe_3_O_4_ composites. As can be seen from the figure, distinctive characteristic peaks appear at 2θ of 30.2°, 35.6°, 43.2°, 57° and 62.6°, corresponding to the five crystal planes (200), (311), (400), (511) and (440) in the Fe_3_O_4_ crystal structure, which proves that the Fe_3_O_4_ nanoparticles are successfully encapsulated in the PS nanofibers.

**Figure 15:**
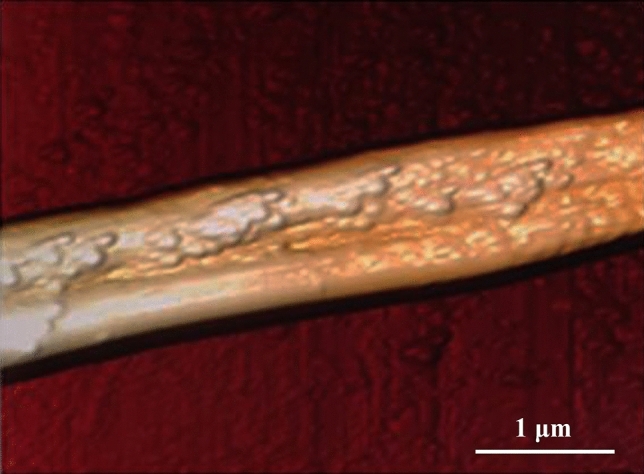
AFM diagram of patterned fibers.

Atomic force microscopy (AFM) shows that the fibers prepared by magnetic spinning have uniform particle size, uniform distribution of Fe_3_O_4_ in the fibers, and good morphology (Fig. 15). Therefore, magnetic nanofibers with good morphology can be obtained by using the MMS process, which provides a new preparation process for the application expansion of functional patterned fibers.

## Conclusions

Using the TRIZ theory of the material field model and conflict matrix, the design method of magnetic spinning device was proposed, a three-dimensional mobile MMS device model was established, a magnetic spinning test prototype was developed, and its performance and influencing factors were experimentally studied, and the following conclusions were drawn.

(1) MMS does not require an external high-voltage power supply and no organic solvent. The magnetic field force can produce sufficient stretching force on the molten polymer fluid to form fibers after overcoming the surface tension, which fundamentally avoids the safety hazards such as electric field breakdown and solvent contamination problems.

(2) The fiber deposition process will not produce a whipping instability phase similar to the electrospinning process, and stable patterned fibers can be obtained. When the collector moves in the plane for linear trajectory (speed 10 mm/s), the oriented fiber with particle size about 10 μm can be obtained, and when the collector moves in the plane for sinusoidal trajectory (speed 20 mm/s), the corresponding sinusoidal pattern fiber with particle size about 900 nm can be obtained.

(3) The morphology of the case fibers is regulated by the trajectory, speed and precision of the moving platform. The additional stretching force generated by the motion of the collector can be used to regulate the fiber particle size.

In summary, the design scheme of the MMS device proposed in this paper is feasible, and the research results provide a new process pathway for the preparation of nanoscale patterned fibers.
